# A Novel Splicing Mutation in the CSF1R Gene in a Family With Hereditary Diffuse Leukoencephalopathy With Axonal Spheroids

**DOI:** 10.3389/fgene.2019.00491

**Published:** 2019-05-22

**Authors:** Xiaodong Yang, Pei Huang, Yuyan Tan, Qin Xiao

**Affiliations:** Department of Neurology, Ruijin Hospital Affiliated to Shanghai Jiaotong University School of Medicine, Shanghai, China

**Keywords:** hereditary diffuse leukoencephalopathy with axonal spheroids, CSF1R, neuroimages, whole-exome sequencing, splicing mutation

## Abstract

Hereditary diffuse leukoencephalopathy with axonal spheroids (HDLS) is a rare autosomal dominant disorder that typically presents with early-onset cognitive decline or personality change. The disease is associated with heterozygous mutations in the colony stimulating factor-1 receptor (CSF1R) gene. CSF1R activation regulates microglial survival, proliferation, and differentiation. The different gene mutations may be related to the various clinical phenotypes. Here, we described comprehensive clinical, neuroimaging, neuropathological, and genetic analyses of a family with HDLS. A novel splicing mutation in intron 13 (c.1858+1G>T) of CSF1R was found in this family. It is located at the splice site of intron 13, resulting in a splice donor site leading to exon 13 skipping from the CSF1R mRNA. The mother and two elderly siblings of the proband had the same CSF1R mutation as the proband but showed very mild neuroimaging abnormalities and mild memory loss, which did not affect daily life, indicating very uneven penetrance and distinctly different disease progression among family members. This report provides diverse neuroimaging and clinical characteristics of a novel CSF1R mutation with different disease penetrance. The large clinical heterogeneity in the same family who all had the same mutation indicates that modifying genes and environmental factors may play a role in the pathogenesis of HDLS.

## Introduction

Hereditary diffuse leukoencephalopathy with axonal spheroids (HDLS) is a rare autosomal dominant disorder characterized by cerebral white matter degeneration and corpus callosum atrophy (Axelsson et al., 1984). It is clinically characterized by adult-onset neuropsychiatric symptoms, progressive cognitive decline, motor and gait disturbances, urinary incontinence, and even seizures (van der Knaap et al., 2000; Wider et al., 2009; Sundal et al., 2012). Neuropathological features of HDLS include a widespread loss of myelin and axons, astrocytosis, and macrophages in the presence of axonal swellings (spheroids) (van der Knaap et al., 2000; Wider et al., 2009). Recently, mutations in the colony stimulating factor 1 receptor (CSF1R) gene were identified as the causative gene of HDLS (Rademakers et al., 2011). To date, more than 60 genetic loci mutations have been linked to HDLS (Guerreiro et al., 2013; Konno et al., 2017; Lynch et al., 2017; Adams et al., 2018; Miura et al., 2018). Most of the mutations were located in the tyrosine kinase domain of the protein (Lynch et al., 2017).

In our present study, we report a novel splicing mutation (c.1858+1G>T) in the CSF1R gene in a family with HDLS. *In vitro* RT-PCR analysis showed that this splice-site mutation resulted in the deletion of exon 13 from mRNA encoded by the CSF1R gene. Our findings expand the molecular and phenotypic spectrum of CSF1R mutation-induced HDLS, which will be useful for screening and the genetic diagnosis of HDLS. The large clinical heterogeneity in the same family who had the same mutation indicates that modifying genes and environmental factors may play a role in the pathogenesis of HDLS.

## Case Presentation

The proband was a 37-year-old man who was referred to our hospital with a 7-month history of progressive weight loss, slurred speech, limb stiffness, and blunt response. Seven months ago, the patient suffered from rapid emaciation with 20-kg weight loss in 2 months. Then, the patient gradually developed dysarthria and occasionally choked when drinking water. Three months later, the patient had upper limb tremors and clumsy hands. Four months later, blunt response, memory loss, and irritability were observed by his family. In the course of the disease, the patient had constipation, accompanied by sweating and sebaceous gland hypersecretion. He denied fever, headache, loss of consciousness, convulsions, muscular atrophy or fibrillation. The medical history information was collected in Feb 2018 when he was admitted to the inpatient department of our hospital. His parents, one elder brother, two elder sisters, and his son are all physically healthy.

On examination, he (high school degree) had a significant global cognitive decline with a mini-mental state examination (MMSE) score of 18/30 and a Montreal cognitive assessment scale (MoCA) score of 15/30. He had obvious dysarthria with bilateral reduced palatal movements, indicating pseudobulbar palsy. Tone was increased in the neck and right limbs. Hyperreflexes of the legs with ankle clonus were noticed, but bilateral pathological signs were negative. His gait had a slow and shuffling characteristic, and he had difficulty turning around. Both upper limbs showed slight postural tremor and clumsy rotation. Finger-nose coordination was slow with mild intention tremor, and poor heel–knee coordination was found on the left limbs.

Blood laboratory tests were normal/negative, including the serum erythrocyte sedimentation rate, C-reactive protein, vitamins (B1\B2\B6\B9\B12), thyroid function, adrenocorticotropic\sex hormone, renin-angiotensin-aldosterone, and antinuclear antibodies. Cerebrospinal fluid analysis of paraneoplastic antibodies, antibodies specific for demyelinating diseases of the central nervous system, autoimmune encephalitis and oligoclonal IgG bands were normal/negative. Syphilis serology and HIV tests were negative.

**Figure 1:**
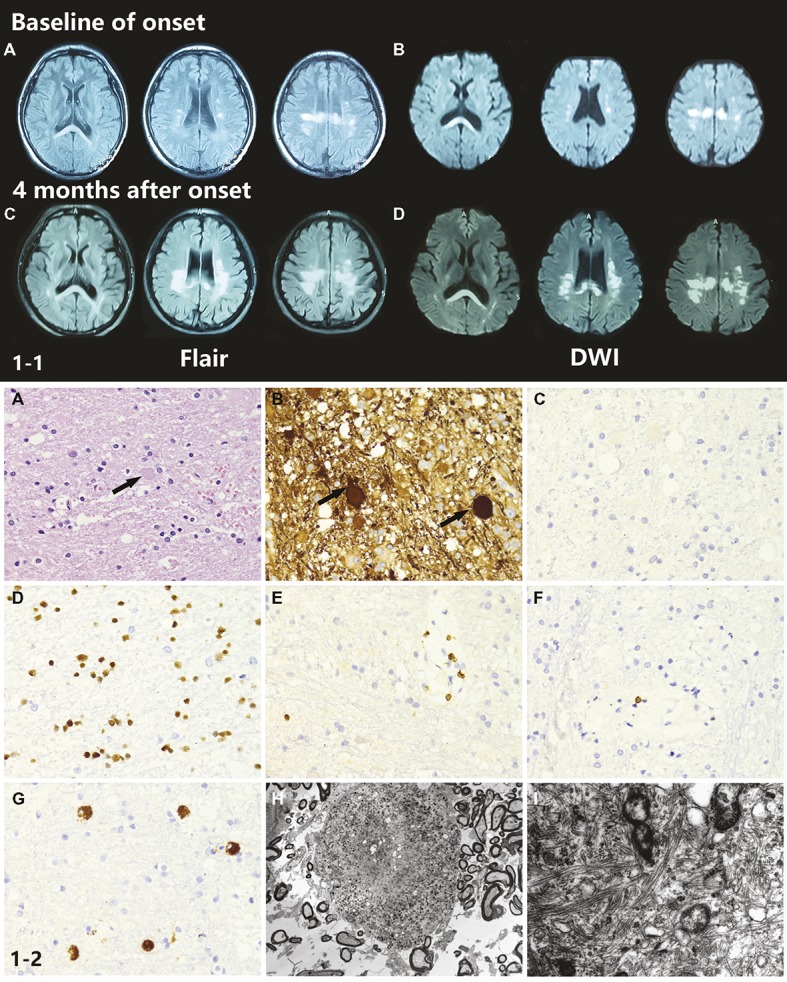
**(1-1)** Neuroimaging findings of the proband. **(A,B)** Brain T2W-Flair and DWI MRI showed multiple, patchy, symmetrical, and hyperintense lesions in periventricular areas, the corpus callosum, and deep white matter regions of the frontal and parietal lobes. The splenium of the corpus callosum showed a homogeneous hyperintense signal in both Flair and DWI. **(C,D)** Four months after onset, the MRI abnormal signal became more widespread and confluent. **(1-2)** Neuropathological findings of the proband. **(A,B)** The H&E stained section of the brain biopsy showed white matter lesions with myelin and axon damage and neuroaxonal swelling (spheroid formation), accompanied by gliosis prominent in the frontal lobe. **(C–G)** The activated microglia and phagocytic cells appeared to be segregated. **(H)** Electron microscopy showed loss of myelin and abnormal axons. Spheroids had thin and discontinuous myelin sheath or no myelin sheath. **(I)** Large numbers of neurofilaments and organelles were observed in the axoplasm.

Brain T2 fluid-attenuated inversion recovery (Flair) and diffusion-weighted imaging (DWI) MRI showed multiple, patchy, symmetrical, and hyperintense lesions in the periventricular areas, corpus callosum, and deep white matter regions of the frontal and parietal lobes. The splenium of the corpus callosum showed a homogeneous hyperintense signal in both Flair and DWI. With disease progression, patchy white matter lesions, which were initially small dots, became more widespread and confluent in this patient. DWI images showed a sustained intense signal over time, which is very unlike DWI high signal in stroke disease. An MRI scan also showed enlarged lateral ventricles (Figures 1-1A–D).

**Figure 2:**
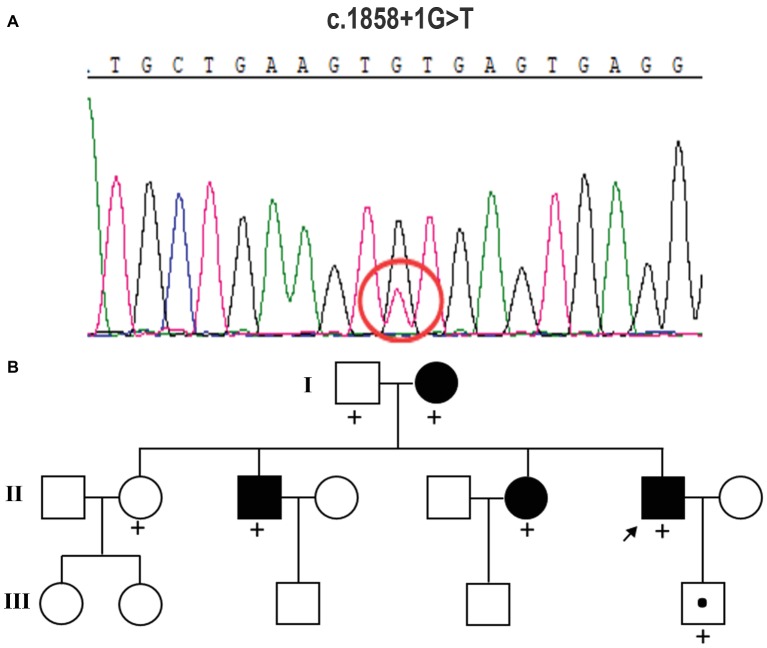
Mutation analysis and identification of the c.1858+1G>T mutation of the CSF1R gene. **(A)** Sequence chromatograms from parts of the CSF1R gene of this case. It displays a splice-site mutation (c.1858+1G>T) in intron 13 of CSF1R. **(B)** Pedigree of the family studied in this report. The affected individuals are indicated with filled squares and circles. The proband is indicated with an arrow. A plus sign indicates that DNA was examined for the CSF1R sequencing analysis.

Histopathological examination of brain biopsy showed white matter lesions with myelin and axon damage and neuroaxonal swelling (spheroid formation), accompanied by prominent gliosis in the frontal lobe (Figures 1-2A,B). The activated microglia and phagocytic cells appeared to be segregated (Figures 1-2C–G). Electron microscopy confirmed the loss of myelin and axons. The axons were swollen and spherical (Figure 1-2H). Spheroids had a thin and discontinuous myelin sheath or no myelin sheath. Large numbers of neurofilaments and organelles were observed in the axoplasm (Figure 1-2I).

## Whole-Exome Sequencing

Peripheral blood samples were collected from the proband, and genomic DNA was extracted from peripheral blood lymphocytes by using an Automated Nucleic Acid Extractor (QIAGEN, Hilden, Germany). Whole-exome sequencing (WES) of the proband was performed by Axeq Technologies (RayLee Biotech, Shanghai, China) using Agilent SureSelect v6 reagents for capturing exons and Illumina HiSeq X Ten platform for sequencing. The percentage of coverage and average depth for WES were 97.86% and 110.5X, respectively. The sequencing reads were aligned to GRCh37.p10 using Burrows-Wheeler Aligner (BWA) software. Reads that aligned to exon regions were collected for mutation identification and subsequent analysis. SAMTools mpileup and bcftools were used to perform variant calling and identify single nucleotide polymorphisms (SNPs) and indels. The called single nucleotide variants (SNVs) and indels were annotated with ANNOVAR. No known variants in the CSF1R gene were found, while the WES test in the proband showed a c.1858+1G>T mutation in exon 13 of CSF1R (Figure 2A), which is a novel mutation of CSF1R. This mutation was not found in the Exome Aggregation Consortium, The Human Gene Mutation Database (HGMD), and the 1,000 Genomes Project (1000G). A gene database analysis using Mutation Taster indicated this mutation as “disease causing.” According to the American College of Medical Genetics and Genomics guideline (Richards et al., 2015), this mutation was predicted to be pathogenic.

**Figure 3:**
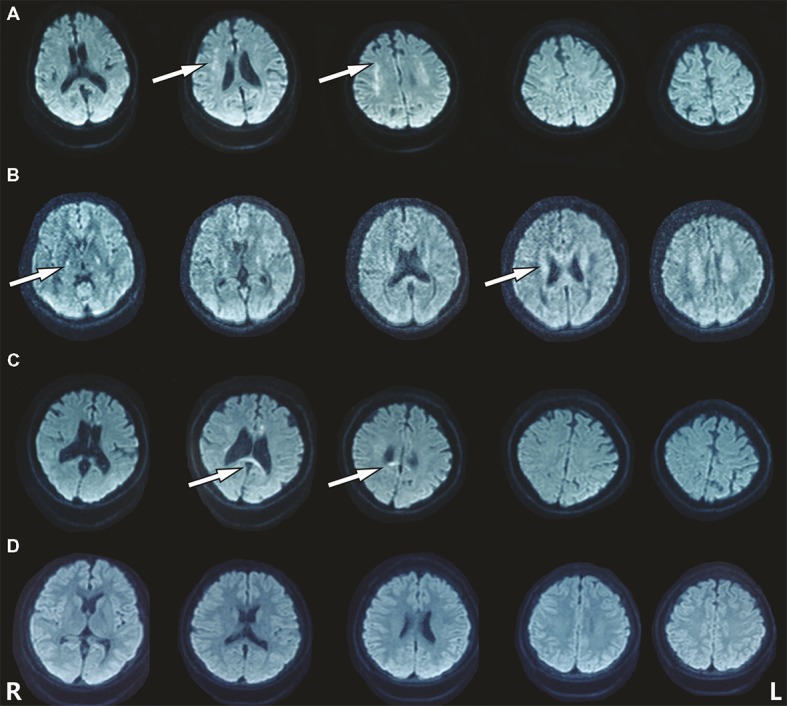
Hyperintense lesions within the white matter were found in the patient’s family members carrying the mutation. **(A)** I:2. **(B)** II:3. **(C)** II:6. **(D)** III:5.

The heterozygous novel mutation identified through WES was verified through Sanger sequencing in all available family members. The same mutation was found in his mother (I:2), brother (II:3), the second elder sister (II:6), and his son (III:5), indicating an autosomal dominant pattern of inheritance (Figure 2B). His mother (age 68), elder brother (age 45), and the second elder sister (age 43) all had complaints of mild memory loss, which did not affect daily life. His 14-year-old son was normal. All of them underwent a 1.5 T MRI scan. Hyperintense lesions within the white matter were found in his mother (Figure 3A), elder brother (Figure 3B), and elder sister (Figure 3C), but much less than this patient, indicating that this mutation had different penetrance among family members. The 14-year-old son had normal brain images so far (Figure 3D). We assumed that he had not yet reach the age of onset.

## Transcription Analysis

**Figure 4:**
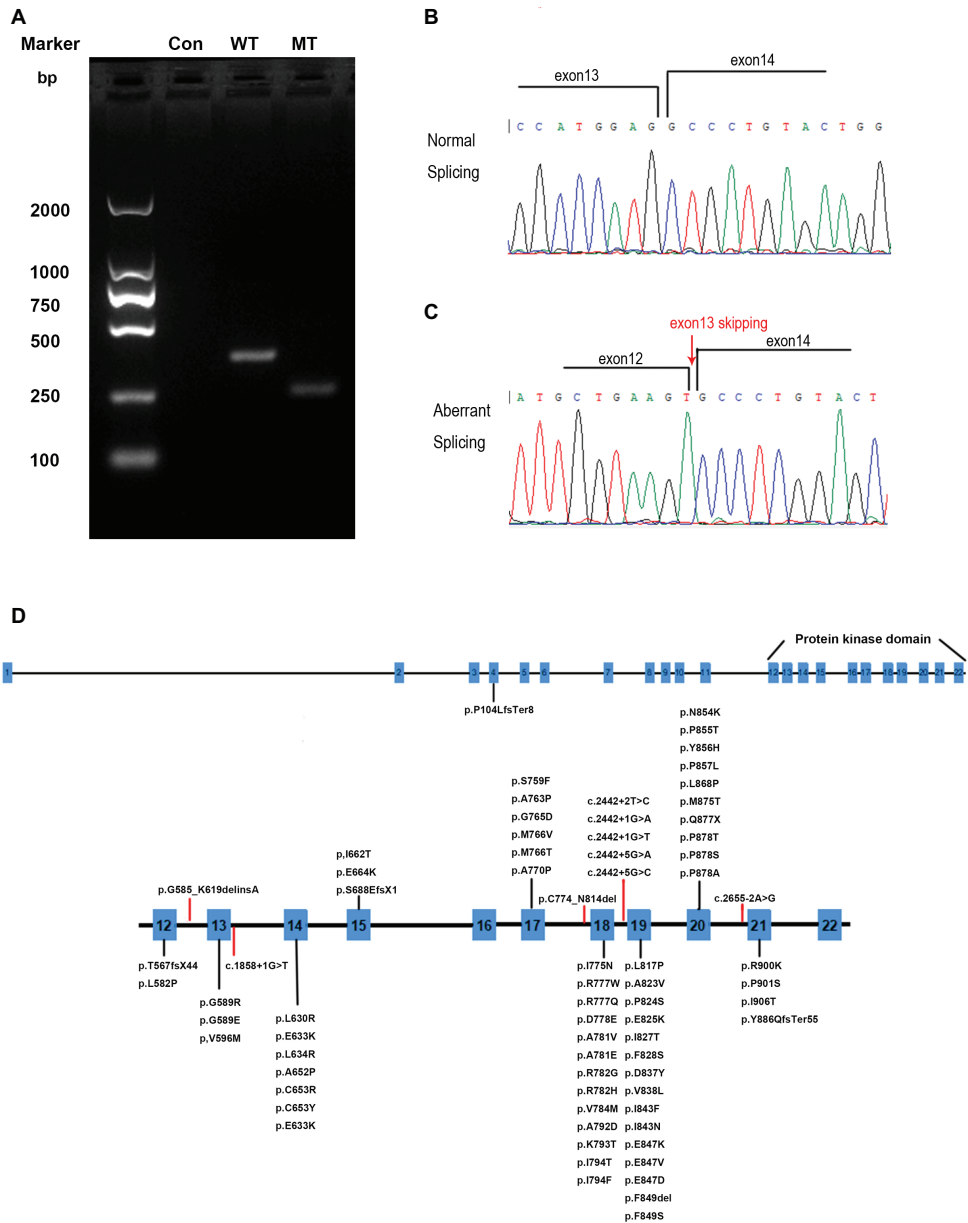
RT-PCR and DNA sequencing results from transfection of wild-type (WT) CSF1R or mutant (MT) c.1858+1G>T CSF1R minigenes. **(A)** Representative agarose gel showing the RT-PCR results of WT, MT, and control minigenes. **(B)** Schematic diagrams identifying the normal splicing transcript (379 bp). **(C)** Schematic diagrams identifying the aberrant splicing transcript with exon 13 (111 bp) skipping are shown. **(D)** CSF1R mutations identified in HDLS. Red lines represent splice-site mutations found in patients with HDLS.

To determine whether the mutation could affect the splicing of mRNA, mutated (MT) CSF1R, which carries the disease-causing c.1858+1G>T mutation and wild-type (WT) CSF1R minigenes, was cloned into the pcDNA3.1 expression vector. pcDNA3.1-CSF1R-WT, pcDNA3.1-CSF1R-MT, and the control pcDNA3.1 (+) plasmids were transiently transfected into 293T cells. We then carried out RT-PCR with primers spanning a region from exon 12 to exon 14 and then separated the products on 2% agarose gels. RNA extracted from cells transfected with wild-type plasmid produced a 340 bp band corresponding to the correct splicing of mRNA, while we detected a truncated product band that was smaller than the wild-type product in cells transfected with the mutated plasmid, thus indicating that an abnormal alternatively spliced isoform was produced by the mutation one base before the splice donor site of exon 13. No band was detected in the cells transfected with pcDNA3.1 (+) control (Figure 4A). The products were purified and confirmed by Sanger sequencing, and sequencing analysis showed that the splice-site mutation caused the deletion of exon 13 of the CSF1R gene during mRNA splicing (Figures 4B,C).

## Discussion

The age of onset of HDLS varies from 15 to 78 years but usually occurs in the third or fourth decade (Wong et al., 2011). Clinical symptoms often start with neuropsychiatric problems, including behavioral changes and cognitive declines, followed by or concurrent with motor and gait disturbances caused by pyramidal, extrapyramidal or cerebellar damage. Resting/postural tremor, urinary, and fecal incontinence also commonly developed (Konno et al., 2017). In the present case, age of onset, dysarthria, motor and gait disorder, cognitive decline, sweating and constipation, and tremors are all typical symptoms of HDLS except the 20-kg weight loss in 2 months without any evidence of loss of appetite, tumor, infection or even vasculitis. Obvious weight loss has never been mentioned in the clinical spectrum of HDLS.

MRI images are marked with bilateral, asymmetrical or symmetrical, patchy or diffuse/confluent T2 hyperintensities along/within periventricular, deep white matter, subcortical of frontal and/or parietal lobes. Diffusion-weighted MRI demonstrates restricted diffusion within white matter lesions that are different from other leukodystrophies, including metachromatic leukodystrophy and X-linked adrenoleukodystrophy. MRI of cerebral autosomal dominant arteriopathy with subcortical infarcts and leukoencephalopathy (CADASIL) might also demonstrate restricted diffusion and diffuse/confluent white matter lesions. However, CADASIL usually has stroke-like episodes, anterior temporal lobe commonly affected, obvious cerebral infarcts, and microbleeding revealed by SWI. In the presented family, the patient exhibited a rapidly progressive trend from patchy white matter lesions to confluent lesions in just 4 months. Other members carried the same CSF1R mutation but had very mild patchy localized lesions corresponding to mild complaints of forgetfulness, indicating very uneven penetrance and quite different disease progression among family members. Some patients with CSF1R mutations might have no or mild symptoms with a very slow disease progression.

The genetic basis of HDLS, mutations in the CSF1R gene on chromosome 5q32, was first elucidated by Rademakers. Functional studies suggested that the mutations affect the kinase activity of the protein, most likely altering the phosphorylation of downstream target (Rademakers et al., 2011). The CSF1R gene encodes a cell-surface receptor containing five immunoglobulin-like domains in the extracellular ligand-binding portion, a single transmembrane domain, and an intracellular tyrosine kinase domain (Pixley and Stanley, 2004). The normal function of CSF1R is essential for proliferation, survival, proliferation, and differentiation of mononuclear phagocytic cells, including microglia in the central nervous system (Stabile et al., 2016). To date, more than 60 genetic loci mutations have been linked to HDLS (Figure 4D) (Guerreiro et al., 2013; Konno et al., 2017; Lynch et al., 2017; Adams et al., 2018; Miura et al., 2018). Almost all of the mutations in CSF1R associated with HDLS are located in the tyrosine kinase domain of the CSF1R protein, encoded by exons 12–21 of the genes (Lynch et al., 2017). Only one frameshift mutation caused by a single nucleotide deletion (c.310delC) in exon 4 resulted in p.Pro104LeufsTer8 located outside of the tyrosine kinase domain (Miura et al., 2018). No disease-associated mutations have been detected in exon 16, and only two have been detected in exon 15 of the gene, suggesting that this domain is not crucial in the pathological pattern of HDLS. Eight splice-site mutations are associated with HDLS, 5 of which are located in intron 18 (Rademakers et al., 2011; Guerreiro et al., 2013; Konno et al., 2014, 2017; Kawakami et al., 2016). The mutation we identified is located at the splice site of intron 13 within the tyrosine kinase domain. RNA splicing is the process during which introns are excised and exons are spliced. These splice donor sites and splice acceptor sites are highly conserved during evolution. The precise recognition of splicing signals is critical to this process. Mutations that affect splicing can cause disease directly or contribute to the severity or susceptibility of disease. It is estimated that approximately 10% of disease-causing mutations affect splicing, and mutations at these splice sites have been found to be associated with a considerable proportion of genetic disorders (Lopez-Bigas et al., 2005; Ward and Cooper, 2010). Splice-site mutations usually lead to abnormal pre-mRNA splicing, which results in exon skipping and activation of cryptic splice sites. Regarding the phenotypic effects of mutations on mRNA splicing, exon skipping occurred more frequently than cryptic splice-site usage (Pridans et al., 2013; van der Klift et al., 2015). From our results, we found that the splice donor site mutation in intron 13 (c.1858+1G>T) of CSF1R caused exon 13 skipping from the CSF1R mRNA, which may further affect the function of the CSF1R protein.

We described detailed clinical, neuroimaging, neuropathological, and genetic analyses of an family with HDLS with different penetrance and disease progression among family members. A novel CSF1R mutation, c.1858+1G>T, located in exon 13, provides an abnormal splice site causing exon 13 to be skipped at the mRNA level, which might lead to abnormal function of the CSF1R protein. Thus far, whether CSF1R mutations result in gain of function, producing dominant negative repressors, or inducing loss of function is controversial. Further research is required to elucidate the detailed molecular mechanism of the CSF1R mutation leading to HDLS.

## Ethics Statement

We obtained written informed consent for genomic analysis of the patient and his family members in accordance with the Declaration of Helsinki. The project was approved by the ethics committee of the Ruijin Hospital Affiliated to Shanghai Jiaotong University School of Medicine. The proband and his family members provided written informed consent for the publication of the patient’s identifiable information.

## Author Contributions

QX conceived and designed the experiments; QX and YT helped in patient workup and recruitment of the patients and their family members; YT helped in genetic analysis; XY and PH performed the experiments and wrote the paper. All authors analyzed and interpreted the data for the study.

### Conflict of Interest Statement

The authors declare that the research was conducted in the absence of any commercial or financial relationships that could be construed as a potential conflict of interest.
